# Mobile phone addiction and achievement motivation in Chinese medical students: the chain mediating effect of anxiety and depression symptoms

**DOI:** 10.3389/fpsyg.2025.1651675

**Published:** 2025-10-29

**Authors:** Haoge Bai, Yiju Wang, Yan Jin, Zongyao Zhang, Juan Wang, Yang Meng, Hao Sun, Hui Xie

**Affiliations:** ^1^Clinical Medical College, Jining Medical University, Jining, Shandong, China; ^2^Department of Psychiatry, National Clinical Research Center for Mental Disorders, and National Center for Mental Disorders, The Second Xiangya Hospital of Central South University, Changsha, Hunan, China; ^3^College of Medical Imaging and Laboratory, Jining Medical University, Jining, Shandong, China

**Keywords:** mobile phone addiction, achievement motivation, anxiety symptom, depressive symptoms, mediating role

## Abstract

**Introduction:**

Mobile phone addiction (MPA) is an escalating issue, particularly among high-pressure medical students prone to over-reliance on mobile phones, and it is closely associated with lower achievement motivation. This study aimed to explore the associations between Chinese medical students' achievement motivation, anxiety, depression, and MPA.

**Methods:**

Single-site cluster sampling was used to recruit 2,977 undergraduate medical students (Years 1–3) from a large medical university in Shandong Province. Data were collected via the Achievement Motivation Scale (AMS), Generalized Anxiety Disorder Scale-7 (GAD-7), Patient Health Questionnaire-9 (PHQ-9), and Mobile Phone Addiction Index (MPAI), with 2,679 valid questionnaires retained.

**Results:**

Of the participants, 26.58% had MPA, with an average MPAI score of 42.97 ± 12.12. The average AMS score was 1.87 ± 0.35. Multiple linear regression showed that gender, liking one's major, MPAI, 7, and PHQ-9 scores significantly influenced achievement motivation (all *p* < 0.05). Mediation analysis indicated that anxiety and depression played a chain mediating role in the relationship between MPA achievement motivation: the total indirect effect was −0.114 (95%CI = [−0.133, −0.095]), including three paths: MPAI → GAD7 → AMS (effect = −0.027, 95%CI = [−0.047, −0.008]), MPAI → PHQ9 → AMS (effect = 95%CI = [−0.042, −0.023]), and MPAI → GAD7 → PHQ9 → AMS (effect = −0.055, 95% CI = [−0.070, −0.041]).

**Discussion:**

MPA correlates with lower achievement motivation in Chinese medical students, with anxiety and depression acting as mediators. Universities should take targeted measures to address MPA, in anxiety and depression, and improve medical students' achievement motivation.

## 1 Introduction

The widespread use of mobile phones has brought considerable convenience, but it has also led to negative consequences such as over-reliance and addiction (Kanar et al., 2022). Mobile Phone Addiction (MPA) refers to an individual's excessive use of mobile phones that cannot be controlled autonomously, resulting in significant impairments in physiological, psychological, and social functioning (Liu et al., 2017). It is sometimes also referred to as “problematic mobile phone use,” “excessive mobile phone use,” or “mobile phone dependence (Li Y. et al., 2020).” According to a 2024 report, the incidence of mobile phone addiction among adolescents is 26.2 % (Cheng et al., 2024). MPA not only disrupts students‘ study plans and time management, causing inattention during classes and procrastination in homework, but also weakens their motivation to learn and self-confidence (Zeerak et al., 2024). Moreover, MPA can have a negative impact on students' social skills and interpersonal interactions, leading to increased avoidance and indifference in real-life social interactions (Shoukat, 2019), thereby exacerbating their feelings of loneliness and helplessness (Zhang and Zeng, 2024) and potentially leading to adverse psychological issues such as anxiety and depression (Li S. et al., 2020).

Achievement motivation is an individual's intrinsic drive to pursue excellence, strive for success, and overcome difficulties to achieve high standards. It reflects an individual's expectation of setting and reaching high-level tasks (McClelland et al., 1953). Atkinson's theory of achievement motivation has been widely used to assess individual performance and explain “why some people are more capable of success than others.” In this theory, achievement motivation is defined as the individual differences resulting from the subtraction of the motivation to avoid failure from the motivation to pursue success (Atkinson, 1957, 1964), and it is used to measure an individual's tendency to strive for success. The tendency to pursue success is influenced and determined by multiple factors of achievement-oriented behavior: (1) the need for success outweighs the need to avoid failure; (2) the expectation of success; and (3) the incentive value of success. Success motivation mainly describes the anticipated pleasure (such as a sense of pride) when achieving a goal, which motivates individuals to engage in situations (Sun et al., 2023). Avoidance of failure motivation mainly describes the discomfort or fear if the goal is not achieved, and these negative emotional expectations cause individuals to resist engaging in situations. Individuals with high achievement motivation prefer more challenging tasks because accomplishing these tasks provides the strongest personal satisfaction (McEwan and Goldenberg, 1999), while those with low achievement motivation tend to show lower levels of academic engagement and fewer attempts at challenging tasks. Research has shown that excessive mobile phone use may have a negative impact on students' achievement motivation (Somayeh et al., 2019).

Medical students face significant academic pressure, as they need to spend a considerable amount of time and energy on their studies and pass rigorous exams. This high-intensity academic workload subjects them to substantial stress. Meanwhile, college students are a group that frequently uses mobile phones. Due to their relatively immature thinking, self-control, and motivation, they are more susceptible to mobile phone addiction (Zhu et al., 2025). MPA can disrupt an individual's normal life, study, and work routines, reduce face-to-face social interactions, and lead to issues in social skills and psychological regulation, thereby triggering anxiety, depression, and increased stress. Anxiety symptoms are generally considered a negative emotion caused by an individual's anticipation of impending danger or disaster, resulting in worry and physical tension (Choe and Yu, 2022). Depression is one of the psychological issues among adolescents, ranging from mild unhappiness to severe sadness, despair, and significant impairment of daily life functions (VandenBos, 2015). Students affected by depression may face various negative outcomes, including drug abuse, academic failure, and even suicide. Therefore, this study focuses on medical students as the research subjects, aiming to regulate their mobile phone addiction through effective guidance and support and to enhance their achievement motivation.

Existing research has demonstrated that MPA is positively correlated with anxiety symptoms, depressive symptoms, fear of missing out, impulsivity, and low achievement motivation (Dong, 2018; Elhai et al., 2017), while it is negatively correlated with high academic performance, positive interpersonal relationships, and high life satisfaction (Lepp et al., 2015). Systematic reviews and meta-analyses have indicated that the severity of MPA is associated with anxiety and depressive symptoms, with a medium effect size, and with stress, with a small to medium effect size (Augner et al., 2023; Hussain et al., 2020; Yang et al., 2020). However, the specific roles of anxiety and depressive symptoms in the relationship between MPA and achievement motivation have not been fully explored. This study aims to investigate the current status of achievement motivation among Chinese medical students and its influencing factors, and further examine the role of anxiety and depression in the relationship between MPA and achievement motivation.

## 2 Methods

### 2.1 Participants and data collection

The study was conducted among all undergraduate medical students (freshmen to juniors) at Jining Medical College, Shandong Province (Nikolic et al., 2023). A cluster sampling method was employed: all classes held between September and December 2024 were randomly selected, and every student in the chosen classes was invited to participate without any additional screening.

Inclusion criteria: (1) full-time medical undergraduate; (2) aged ≥ 18 years; (3) daily mobile-phone use ≥ 1 h; (4) provided written informed consent. Exclusion criteria: (1) self-reported severe neurological or psychiatric disorder; (2) non-medical major; (3) > 20 % questionnaire items missing.

Investigators received standardized training on scale administration. Written informed consent was obtained immediately before questionnaire distribution. Completed forms were checked on the spot; missing responses were supplemented when possible. A total of 2,977 students were approached and 2,679 valid questionnaires were retained (effective response rate = 90.0%). The study protocol was approved by the Research Ethics Committee of Jining Medical University.

### 2.2 Measurements

#### 2.2.1 Basic demographic characteristics

The basic demographic characteristics assessed in this questionnaire survey included gender, age, whether the respondent was an only child, whether the respondent had experienced being left-behind before the age of 18, health status (of the respondent, father, and mother), monthly living expenses, annual family income, father's educational level, mother's educational level, whether the respondent liked their major, whether they would choose the same major again, and their career goals after graduation. The specific variable coding is presented in Supplementary material S1.

#### 2.2.2 Patient health questionnaire-9(PHQ-9)

The PHQ-9 is a depression symptom screening questionnaire developed by Kroenke et al. (2001) and has demonstrated good reliability and validity across various populations. The scale consists of nine items, each with four response options, scored on a 0–3 scale. The total score ranges from 0 to 27, with higher scores indicating more severe depressive symptoms. Scores from 0 to 4 are interpreted as no depression, while scores of 5 or higher indicate the presence of depressive symptoms. Specifically, scores ranging from 5 to 9 are classified as mild depression, and scores of 10 or above are considered moderate or more severe depression (Levis et al., 2019). In this study, the Chinese version translated by Wang et al. (2014) was used (see Supplementary material S2). The standardized Cronbach's alpha coefficient for this scale in this study was 0.857, indicating good internal consistency. For more detailed information, refer to Supplementary material S6, S7.

#### 2.2.3 Generalized anxiety disorder scale-7(GAD-7)

The GAD-7 is a 7-item self-assessment scale developed by Spitzer, Kroenke, and Williams in 2006 based on DSM-IV criteria (Spitzer et al., 2006), designed to evaluate the severity of Generalized Anxiety Disorder (GAD). The scale consists of seven items, each with four response options scored a on 0–3 scale. The total score ranges from 0 to 21, with higher scores indicating more severe anxiety symptoms. Scores from 0 to 4 are considered to indicate no or minimal anxiety (i.e., anxiety without clinical significance), 5–9 to indicate mild anxiety, 10–14 to indicate moderate anxiety, and 15 or above to indicate severe anxiety (Sapra et al., 2020). A total score of ≥10 may suggest the presence of Generalized Anxiety Disorder (GAD). In this study, the Chinese version translated by He Tieyan and Li Chun was used (see Supplementary material S3). The standardized Cronbach's alpha coefficient for this scale in this study was 0.898, indicating good internal consistency. For more detailed information, refer to Supplementary material S6, S8.

#### 2.2.4 Mobile phone addiction index (MPAI)

The Mobile Phone Addiction Inventory (MPAI) was revised and streamlined by Leung based on the Mobile Phone Problem Use Scale (MPPUS) (Leung, 2008). It is a widely recognized tool for assessing the severity of mobile phone addiction and has been extensively used among adolescents and student populations. The MPAI consists of 17 items across four dimensions: Inability to Control Craving, Anxiety and Feeling Lost, Withdrawal and Escape, and Productivity Loss. Each item is rated on a 5-point Likert scale ranging from “not at all” (1 point) to “always” (5 points), with total scores ranging from 17 to 85. Higher scores indicate a higher degree of mobile phone addiction. Typically, a total score above a certain threshold (e.g., 50 points) is considered to be within the clinical range of mobile phone addiction (An et al., 2022). In this study, the Chinese version developed by Professor Liang Yongzhi in 2008 was used (see Supplementary material S4). The standardized Cronbach's alpha coefficient for this scale in this study was 0.883, indicating good internal consistency. For more detailed information, refer to Supplementary material S6, S9.

#### 2.2.5 Achievement motivation scale (AMS)

The Achievement Motivation Scale (AMS) was originally developed by Norwegian psychologists Gjesme, T. and Nygard, R. in 1970 and later translated into Chinese by Ye and Kunt in 1992 (Ye and Kunt, 1992). It is widely used to measure the intrinsic motivation of individuals when pursuing success and achievement. The scale consists of two dimensions: Motivation to Succeed (Ms) and Motivation to Avoid Failure (Mf), each comprising 15 items. Each item is scored on a 1–4 scale with four response options. The total scores for Ms and Mf are calculated separately, and the overall achievement motivation score is obtained by subtracting Mf from Ms (Peng and Zhang, 2024). A score of Ms-Mf > 0 indicates strong achievement motivation; Ms-Mf = 0 indicates moderate achievement motivation; and Ms-Mf < 0 indicates weak achievement motivation. The Chinese version used in this study is provided in Supplementary material S5. The standardized Cronbach's alpha coefficient for this scale in this study was 0.767, indicating good internal consistency. For more detailed information, refer to Supplementary material S6, S10.

### 2.3 Data analysis

In this study, data entry and cleaning were conducted using Epi Data. After double-checking by two individuals, the data were analyzed using SPSS 26.0 software. Descriptive statistics were presented as Mean (SD) for scale scores and as frequency (percentage) for demographic variables. In view of the large sample size (all cells n > 100), absolute skewness ≤ 3 and kurtosis ≤ 10 were taken as evidence of approximate univariate normality (Kline, 2015). Levene's test for equality of variances was used to assess whether the variances were equal across groups. Independent samples t-tests and ANOVA were employed to examine differences in AMS scores among groups, for ANOVA, *post-hoc* pairwise comparisons were carried out with LSD when variances were equal or Tamhane's T2 when variances were unequal. Pearson correlation analysis was used to assess the relationships between AMS scores and other scale scores. Variables with statistically significant differences in demographic characteristics, as well as PHQ-9, GAD-7, and MPAI, were included in multiple linear regression analysis. The assumptions of this analysis—linearity, independence, homoscedasticity, multicollinearity (VIF < 10), and normality of residuals—were examined and met. Parallel mediation effect analysis was conducted using Model 4 of the PROCESS macro. A significance level of *p* < 0.05 was applied to all statistical tests.

## 3 Results

### 3.1 Common method bias test

The Harman single-factor test was employed to assess common method bias. The results indicated that there were five factors with eigenvalues greater than 1, and the variance explained by the first common factor was 18.42%, which is below the critical value of 40%. Therefore, it can be concluded that there is no significant common method bias in the data of this study.

Correlation analysis was conducted among four variables: depression (PHQ-9), anxiety (GAD-7), mobile phone addiction (MPAI), and achievement motivation (AMS). The results showed that there were significant correlations among anxiety, depression, mobile phone addiction, and achievement motivation (p < 0.01), as presented in Table 1.

**Table 1:** Correlation analysis of study variables.

**Variable**	**PHQ-9**	**GAD-7**	**MPAI**	**AMS**
PHQ-9	1.000			
GAD-7	0.739^**^	1.000		
MPAI	0.418^**^	0.390^**^	1.000	
AMS	−0.402^**^	−0.353^**^	−0.399^**^	1.000

^**^p < 0.01.

### 3.2 Mediation analysis and effect decomposition

To further explore the underlying mechanisms between Mobile Phone Addiction Inventory (MPAI) scores and Achievement Motivation Scale (AMS) scores, mediation analysis was conducted, incorporating factors with statistically significant differences identified in the previous analyses (Gender, Left-behind experience before 18 years old, Like own professional, Will choose own professional again, Family annual income, Father's educational level, Mother's educational level, Goals after graduation) as covariates.

**Figure 1:**
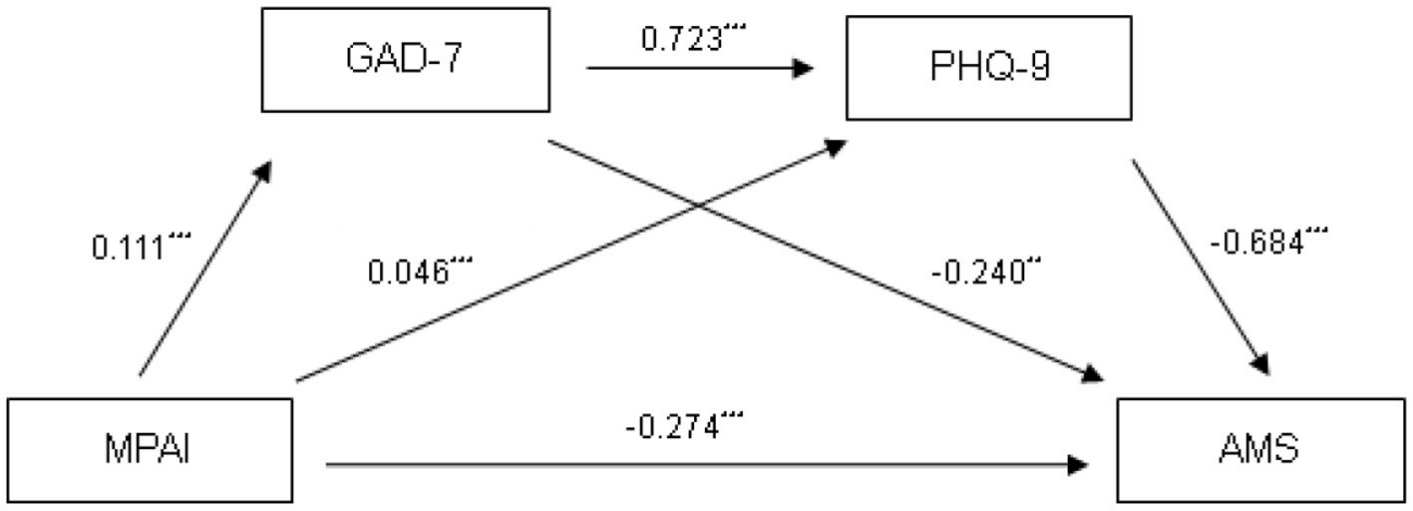
Parallel-mediation model. ^**^p < 0.01. ^***^p < 0.001.

Table 2 presents the results of the mediation analysis. The direct effect of MPAI scores on AMS scores, as well as the indirect effects mediated through Generalized Anxiety Disorder (GAD-7) scores and Patient Health Questionnaire (PHQ-9) scores, were examined. The confidence intervals (CIs) for both the direct and indirect paths did not include zero, indicating significant relationships. Specifically, the total direct effect of MPAI scores on AMS scores was −0.274, with a 95% CI of [−0.312, −0.237]. The total indirect effect was −0.114, with a 95% CI of [−0.133, −0.095]. The specific indirect paths were as follows: the path mediated through GAD-7 scores had an effect of −0.027 (95% CI = [−0.047, −0.008]), the path mediated through PHQ-9 scores had an effect of −0.032 (95% CI = [−0.042, −0.023]), and the chained mediation effect through both GAD-7 and PHQ-9 scores had an effect of −0.055 (95% CI = [−0.070, −0.041]). These effects accounted for 6.96, 8.25, 14.17, 10.56, and 11.76% of the total effect (total effect = −0.388; 95% CI = [−0.423, −0.353]), respectively. These results suggest that GAD-7 and PHQ-9 scores may be important psychological pathways through which MPAI scores are associated with AMS scores. The detailed path model is illustrated in Figure 1.

**Table 2:** Direct, indirect, and total effects of the parallel-mediation model (*N* = 2,679).

**Model pathway**	**β**	**SE**	**95% CI (bootstrap test)**	**Proportion of mediating effect**	***P***
			**[Lower, Upper]**		
Total effect	−0.388	0.018	−0.423, −0.353	100%	<0.001
Total direct effect	−0.274	0.019	−0.312, −0.237	67.50%	<0.001
Total indirect effect	−0.114	0.010	−0.133, −0.095	35.29%	<0.001
MPAI → GAD7 → AMS	−0.027	0.010	−0.047, −0.008	12.94%	<0.001
MPAI → PHQ9 → AMS	−0.032	0.005	−0.042, −0.023	10.56%	<0.001
MPAI → GAD7 → PHQ9 → AMS	−0.055	0.007	−0.070, −0.041	11.76%	<0.001

### 3.3 Reliability and validity test of the scale

The reliability and validity of the four scales were examined (Supplementary material S6–S10). Overall, internal consistency was excellent (Cronbach's α 0.83–0.91), convergent validity satisfactory (AVE 0.49–0.89), and all standardized loadings >0.50 (*p* < 0.001), indicating sound psychometric properties.

### 3.4 Demographic characteristic

The demographic characteristics of the participants were statistically described in this study. Specifically, there were 1,156 males (43.15%) and 1,523 females (56.85%). In terms of age, 1,278 participants were 18 years old or younger (47.70%), 559 were 19 years old (20.87%), 575 were 20 years old (21.46%), and 267 were 21 years old or older (9.97%). Regarding family structure, 749 participants were only children (27.96%), and 1,930 were not only children (72.04%). For more detailed demographic information, see Tables 3, 4.

**Table 3:** Independent sample *t*-test of AMS score.

**Item**	**Option**	***N* (%)**	**Score of AMS (SD)**	***t***	***P* (uncorrected)**	**Bonferroni significance (α = 0.010)**
Gender	Male	1,156 (43.15%)	3.30 (12.49)	5.240	<0.001	<0.001
	Female	1,523 (56.85%)	0.78 (12.13)			
Single son or daughter	Yes	749 (27.96%)	2.63 (13.34)	1.981	0.048	0.24
	No	1,930 (72.04%)	1.57 (11.93)			
Left-behind experience before 18 years old	Yes	445 (16.61%)	−0.77 (12.31)	−4.958	<0.001	<0.001
	No	2,234 (83.39%)	2.39 (12.29)			
Like own professional	Yes	2,495 (93.13%)	2.30 (12.20)	6.709	<0.001	<0.001
	No	184 (6.87%)	−3.98 (12.89)			
Will choose own professional again	Yes	2,179 (81.34%)	2.60 (12.16)	6.441	<0.001	<0.001
	No	500 (18.66%)	−1.32 (12.68)			

Bonferroni correction was applied (k = 5 comparisons); threshold α = 0.010; *p* < 0.010 indicates statistical significance.

**Table 4:** ANOVA of AMS score.

**Item**	**Option**	***N* (%)**	**Score of AMS(SD)**	***F***	***P***
Age	18 years and under	1,278 (47.70%)	2.08 (11.99)	1.240	0.293
	19 years	559 (20.87%)	2.34 (12.69)		
	20 years	575 (21.46%)	1.42 (12.58)		
	21 years and older	267 (9.97%)	0.85 (12.77)		
Monthly cost of living	0–1,000 Yuan	202 (7.54%)	1.54 (12.51)	0.747	0.524
	1,001–1,500 Yuan	1,226 (45.76%)	1.54 (11.63)		
	1,501–2,000 Yuan	1,044 (38.97%)	2.19 (12.82)		
	2,001 Yuan and above	207 (7.73%)	2.49 (13.82)		
Family annual income	0–30,000 Yuan	529 (19.75%)	0.55 (12.04)	6.340	<0.001
	30,001–50,000 Yuan	451 (16.83%)	0.72 (11.71)		
	50,001–80,000 Yuan	340 (12.69%)	1.03 (12.21)		
	80,001–1,00,000 Yuan	395 (14.74%)	1.64 (12.59)		
	1,00,001–1,50,000 Yuan	543 (20.27%)	2.96 (11.76)		
	1,50,001 Yuan and above	421 (15.71%)	4.24 (13.58)		
Father's educational level	Primary school and below	232 (8.66%)	1.02 (11.62)	3.236	0.006
	Junior high school	911 (34.01%)	1.06 (12.07)		
	High school	547 (20.42%)	2.02 (12.01)		
	College	469 (17.51%)	1.68 (12.64)		
	Undergraduate	441 (16.46%)	3.68 (12.47)		
	Master's degree and above	79 (2.95%)	3.56 (16.03)		
Mother's educational level	Primary school and below	399 (14.89%)	−0.11 (12.22)	3.634	0.003
	Junior high school	905 (33.78%)	2.17 (11.90)		
	High school	483 (18.03%)	1.54 (12.04)		
	College	471 (17.58%)	1.89 (12.96)		
	Undergraduate	380 (14.18%)	3.48 (12.43)		
	Master's degree and above	41 (1.53%)	3.15 (16.37)		
Goals after graduation	No direction	39 (1.46%)	−3.87 (15.59)	6.137	<0.001
	Employment	273 (10.19%)	−0.64 (12.17)		
	Civil servants	74 (2.76%)	2.73 (12.70)		
	Postgraduate	2,279 (85.07%)	2.21 (12.21)		
	Other	14 (0.52%)	7.14 (17.14)		

### 3.5 Descriptive statistics of scale scores

**Figure 2:**
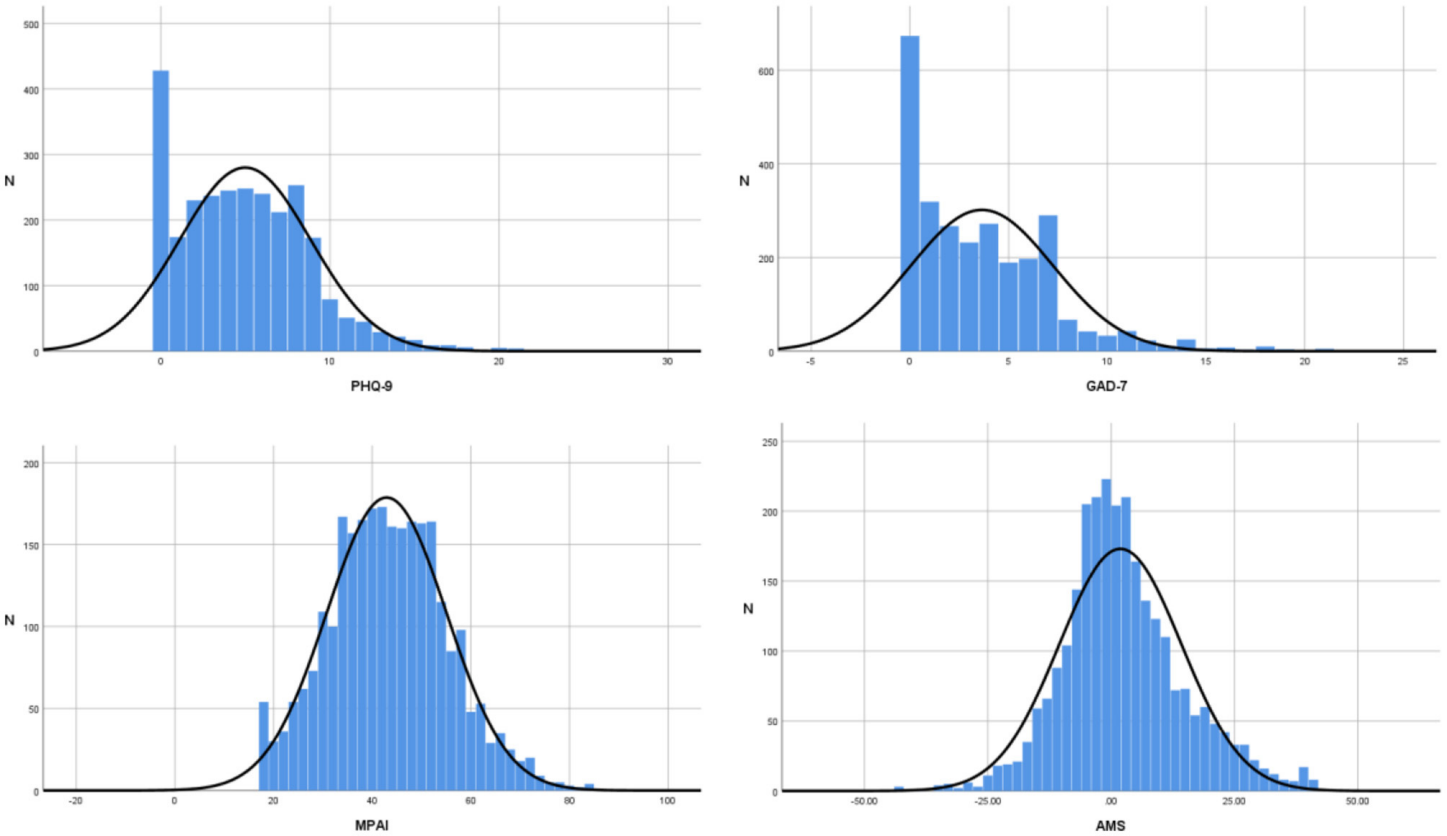
Score histogram of each scale.

The scores of the scales involved in this study were statistically described using SPSS 26.0 software: PHQ-9 (4.99 ± 3.88), GAD-7 (3.66 ± 3.60), MPAI (42.97 ± 12.12), and AMS (1.87 ± 12.35). Histograms of the scale scores are presented in Figure 2. Normality of the AMS total score was examined in SPSS 26.0 for the overall sample (*N* = 2 679). The distribution exhibited a skewness of 0.375 (SE = 0.047) and a kurtosis of 0.737 (SE = 0.095). Following Kline (2015) guidelines for large samples (|skewness| ≤ 3, |kurtosis| ≤ 10), these values indicate an approximately normal distribution, satisfying the assumption for parametric analyses.

### 3.6 Independent sample *t*-test and ANOVA

Tables 3, 4 summarize AMS-score differences among medical-student subgroups. After Bonferroni correction (*k* = 5, α = 0.010), only-child status was no longer significant. Male students, those who liked their major and those willing to re-choose it reported significantly higher AMS scores (*p* < 0.001), whereas students with left-behind experience before age 18 scored significantly lower (*p* < 0.001). One-way ANOVA showed no significant differences by age or monthly living expenses; however, scores differed significantly across categories of annual family income, father's education, mother's education and post-graduation goals.

### 3.7 *Post hoc* Multiple comparisons of one-way ANOVA

The results of multiple comparisons showed that for family annual income, the AMS scores of the group with an income of 150,001 Yuan and above were significantly lower than those of the groups with incomes of 10,001–30,000 Yuan (*p* = 0.001), 30,001–50,000 Yuan (*p* < 0.001), 50,001–80,000 Yuan (*p* = 0.024), and 80,001–100,000 Yuan (*p* = 0.003). The AMS scores of the group with an income of 100,001–150,000 Yuan were significantly lower than those of the groups with incomes of 10,001–30,000 Yuan (*p* = 0.004) and 30,001–50,000 Yuan (*p* < 0.001).

For father's educational level, the AMS scores of children whose fathers had a college degree or above were significantly higher than those whose fathers had primary school or below (*p* = 0.008), junior high school (*p* < 0.001), and high school (*p* = 0.036). The AMS scores of children whose fathers had a master's degree or above were significantly higher than those whose fathers had junior high school (*p* = 0.004).

For mother's educational level, the AMS scores of children whose mothers had primary school or below were significantly lower than those whose mothers had junior high school (*p* = 0.002), high school (*p* = 0.047), and associate degree (*p* = 0.017). The AMS scores of children whose mothers had a college degree or above were significantly higher than those whose mothers had primary school or below (*p* < 0.001), and high school (*p* = 0.022).

For post-graduation goals, the AMS scores of the postgraduate entrance examination group were significantly higher than those of the no-direction group (*p* = 0.002) and the employment group (*p* < 0.001). The AMS scores of the civil servant examination group were significantly higher than those of the no-direction group (*p* = 0.007) and the employment group (*p* = 0.036). The AMS scores of the employment group were significantly higher than those of other groups (*p* = 0.021).

For more detailed results, refer to Supplementary material S11.

### 3.8 Multivariate linear regression analysis of AMS scores of medical students with different characteristics

A multiple linear regression analysis was conducted with AMS scores as the dependent variable, incorporating variables with statistically significant differences in the demographic characteristics analysis, as well as scores from the PHQ-9, GAD-7, and MPAI. The results indicated that gender, liking one's major, PHQ-9 scores, GAD-7 scores, and MPAI scores had statistically significant effects on AMS scores. Model fit: *R*^2^ = 0.18, *F*_(23, 2655)_ = 22.3, *p* < 0.001; residuals were normal, homoscedastic, and no severe multicollinearity was present (all VIF < 2), satisfying the assumptions of multiple linear regression (see Table 5 footnote for details).

**Table 5:** Multiple linear regression analysis of AMS scores of medical students with different characteristics.

**Items**	**Options**	**B**	**SE**	***t***	***P***
Gender	Male	1.935	0.421	4.591	<0.001
	Female (control group)	0			
Left-behind experience before 18 years old	Yes	−0.292	0.583	−0.500	0.617
	No (control group)	0			
Like own professional	Yes	2.278	0.898	2.537	0.011
	No (control group)	0			
Will choose own professional again	Yes	1.037	0.580	1.788	0.074
	No (control group)	0			
Family annual income	3,0001–50,000 Yuan	−0.512	0.690	−0.742	0.458
	50,001–80,000 Yuan	−0.411	0.754	−0.545	0.586
	80,001–1,00,000 Yuan	0.109	0.727	0.150	0.881
	1,00,001–1,50,000 Yuan	1.180	0.688	1.717	0.086
	1,50,001 Yuan and above	2.105	0.754	2.792	0.005
	0–30,000 Yuan (control group)	0			
Father's educational level	Junior high school	−0.757	0.819	−0.924	0.355
	High school	−0.972	0.915	−1.062	0.288
	College	−1.182	1.001	−1.181	0.238
	Undergraduate	−0.362	1.071	−0.338	0.735
	Master's degree and above	0.033	1.623	0.020	0.984
	Primary school and below (control group)	0			
Mother's educational level	Junior high school	1.131	0.675	1.675	0.094
	High school	0.160	0.814	0.196	0.844
	College	0.711	0.890	0.798	0.425
	Undergraduate	1.019	0.992	1.027	0.305
	Master's degree and above	1.106	2.009	0.550	0.582
	Primary school and below (control group)	0			
Goals after graduation	Employment	3.998	1.845	2.167	0.030
	Civil servants	7.150	2.129	3.358	0.001
	Postgraduate	5.827	1.743	3.344	0.001
	Other	6.581	3.356	1.961	0.050
	No direction (control group)	0			
Score of PHQ-9	-	−0.676	0.082	−8.283	<0.001
Score of GAD-7	-	−0.240	0.087	−2.771	0.006
Score of MPAI	-	−0.277	0.019	−14.44	<0.001

Model summary: *R*^2^ = 0.18, adjusted *R*^2^ = 0.17, *F*_(23, 2, 655)_ = 22.3, *p* < 0.001. Assumption checks: multicollinearity (all VIF < 2), residual normality (Shapiro-Wilk *p* = 0.08), homoscedasticity (Breusch-Pagan *p* = 0.12), independence (Durbin-Watson = 1.97).

## 4 Discussion and limitations

### 4.1 Discussion

Achievement motivation is closely related to mental health (Varghese et al., 2015). In this study, Chinese medical students obtained a mean AMS score of 1.87. Multiple linear regression showed that being male and liking one's major were associated with higher AMS scores, whereas higher PHQ-9, GAD-7, and MPAI scores were associated with lower AMS scores (Shahroudi et al., 2019).

#### 4.1.1 Mobile phone addiction and achievement motivation

In this study, the degree of mobile phone addiction was measured by the MPAI score. The results of multiple linear regression analysis show that higher MPAI scores are associated with lower levels of achievement motivation, consistent with the findings of Yaghoobi et al. (2024). Mobile phone addiction is significantly correlated with students' anxiety and depressive symptoms. When individuals spend a large amount of time and energy on mobile phones, they are unable to focus on achieving their goals, which weakens their achievement motivation (Ye et al., 2025). Moreover, individuals with mobile phone addiction often fail to complete tasks or achieve goals due to excessive phone use, leading to a sense of frustration. They gradually begin to doubt their own abilities, forming a negative cognitive pattern of “I can't do it,” which results in low self-efficacy and weakens their achievement motivation (Ran, 2022). Building on the evidence of the negative impact of mobile phone addiction on achievement motivation, this study further explored the mediating role of anxiety and depressive symptoms in the relationship between mobile phone addiction and weakened achievement motivation. The results show that anxiety and depressive symptoms play a significant mediating role in this relationship, exhibiting a chain mediation effect pattern. Mobile phone addiction is not only directly negatively correlated with achievement motivation but also exacerbates its negative impact on achievement motivation by causing anxiety and depressive symptoms (Amin et al., 2024). The specific mechanism may be that mobile phone addiction causes medical students to become immersed in the virtual world (Zhang et al., 2022), gradually detaching from real academic and social contexts, thereby weakening their achievement motivation. Additionally, academic challenges and interpersonal pressures in reality, which should be important stimuli for enhancing achievement motivation, are often avoided by students who are overly reliant on mobile phones. This avoidance leads to a lack of motivation and confidence when facing academic tasks and generates anxiety and depressive symptoms (Chen et al., 2024). These negative emotions further intensify the avoidance tendency, making it more difficult for students to break free from mobile phone addiction and creating a vicious cycle (Nikolic et al., 2023).

#### 4.1.2 Gender and achievement motivation

Male medical students had significantly higher AMS scores than females, suggesting that “masculinity may be a key structure differentiating individual achievement motivation.” Gender and gender roles are social constructs that reflect societal expectations and norms regarding the roles, behaviors, activities, and characteristics of men and women. These constructs are shaped by multifaceted factors, including sociocultural contexts, education, and family influences (Cunha-Oliveira et al., 2021). Families may have different expectations and support mechanisms for male and female children. Some families may place higher expectations on male children and provide them with more attention and resource support, which can enhance their sense of encouragement and motivation, thereby strengthening their achievement motivation. Research by Zhan et al. (2023) has shown significant differences in parental expectations for boys and girls in terms of expression, thinking, knowledge acquisition, concentration, and hands-on abilities. Additionally, fathers and mothers may have different expectations for their children's resilience. In terms of educational resource allocation, male students are more likely to access high-quality educational resources and learning opportunities compared to female students (Arnold and Fox, 2025), such as participating in various medical experiments, medical competitions, and research projects (Ladachart and Ladachart, 2025). These resources and opportunities can help male students enhance their capabilities and confidence, which in turn strengthens their achievement motivation.

#### 4.1.3 Professional identity and achievement motivation

Professional identity, derived from the psychological concept of “ego identity,” reflects an individual's affirmation of their chosen profession and is manifested in positive attitudes such as job satisfaction. It is an important basis for studying individual career development (Holland et al., 1993). The results of the multiple linear regression analysis in this study show that medical students who like their major have higher AMS scores. The higher the professional identity of medical students, the more confident they are in completing challenging tasks, the more satisfaction they derive from their academic and professional lives, and the more motivated they are to tackle various challenges, leading to stronger achievement motivation (Chen, 2023). When college students have a high level of identification with their major, they are more likely to actively engage in learning and practice, willing to invest more effort and time in professional challenges. This positive attitude helps enhance their professional skills and knowledge, thereby boosting their self-confidence and sense of self-efficacy, which in turn enables them to exhibit stronger achievement motivation when facing difficult tasks. Universities should place greater emphasis on cultivating students' professional identity by employing various approaches and methods to help students develop a positive cognitive and emotional connection with their major, thereby stimulating their learning motivation and achievement motivation and promoting their comprehensive development and growth.

#### 4.1.4 Anxiety symptoms, depressive symptoms, and achievement motivation

Anxiety and depression are frequent negative emotional states among medical students and have been repeatedly shown to impair cognitive function, learning strategy and academic persistence (Bandelow and Reitt, 2015; Kessler et al., 2020). Anxiety is characterized by excessive worry and nervousness about the future, while depression involves negative evaluations of the present and a sense of hopelessness about the future (American Psychiatric Association., 2022). In line with previous work (e.g., Putwain and Daly, 2021), our results showed that higher levels of anxiety (GAD-7) and depression (PHQ-9) were associated with lower achievement-motivation scores. Students reporting high anxiety/depression are more likely to experience academic withdrawal, reduced perseverance and diminished striving for excellence. Therefore, universities should implement comprehensive measures to support the mental health of medical students, helping them overcome psychological barriers and improve their academic performance and job satisfaction. Medical students themselves should also actively manage their mental health and cultivate good coping abilities to better face the challenges in their academic and professional lives. Through joint efforts, the adverse effects of anxiety and depression on medical students can be effectively mitigated, promoting their comprehensive development and growth (Zhang et al., 2022).

### 4.2 Implications

This study has validated the factors influencing achievement motivation among Chinese medical students and constructed a chain mediation model of “Mobile Phone Addiction → Anxiety Symptoms → Depressive Symptoms → Low Achievement Motivation,” revealing the critical role of anxiety and depressive symptoms in the relationship between mobile phone addiction and achievement motivation (Putwain and Daly, 2021). This finding not only enriches the theoretical understanding of the relationship between mobile phone addiction and achievement motivation but also provides practical suggestions for medical students, educators, psychological counselors, and parents. Medical students should rationally recognize the dangers of mobile phone addiction, take proactive measures to prevent it, and actively engage in academic supervision (Meng et al., 2025). Schools should place high importance on students' mental health by offering mental health education courses and providing professional psychological counseling to help students effectively cope with negative emotions and enhance their psychological resilience (Xue et al., 2024; Hatice and Yuksel, 2016). Additionally, teachers should actively create a lively classroom atmosphere, optimize course settings, improve teaching methods, and increase classroom engagement to provide students with positive emotional experiences and a sense of achievement during the learning process, thereby further strengthening their motivation to pursue success. Importantly, the chain mediation path explains 42 % of the variance in achievement motivation, indicating that interventions targeting any link—reducing phone addiction, alleviating anxiety, or mitigating depressive symptoms—are likely to yield measurable gains in students' academic drive (Chen et al., 2024).

### 4.3 Limitations

The current study has achieved significant results, yet it also has some limitations that should be addressed in future research. The sample in this study was limited to a single medical college in Jining, Shandong Province, China. The narrow geographical scope and the specific type of institution may restrict the generalizability of the findings. Future research should consider expanding the sample to include medical schools from different regions and at various levels to enhance the representativeness of the conclusions.

Moreover, the cross-sectional design of this study limits the ability to establish causal relationships between the variables. Subsequent research could employ longitudinal cohort study designs to observe the developmental trajectories of mobile phone addiction, anxiety, depression, and achievement motivation over an extended period. This approach would provide a deeper understanding of the causal relationships and offer more compelling theoretical support for targeted interventions.

Additionally, the precise nature of mobile content consumed by participants was not assessed in the present study. We intend to incorporate comprehensive content-use items in future work to disentangle these potential differential effects.

## Data Availability

The raw data supporting the conclusions of this article will be made available by the authors, without undue reservation.
