# Supplementary material for: Mobile phone addiction and achievement motivation in Chinese medical students: the chain mediating effect of anxiety and depression symptoms
Source: Front Psychol. 2025 Oct 29;16:1651675. doi: 10.3389/fpsyg.2025.1651675 (PMC12605222; doi:10.3389/fpsyg.2025.1651675)
Supplement: Supplementary file 1 [file Table_1.docx]

Supplementary Materials

S1 Variable assignment

| Variable | Assignment |
| --- | --- |
| *Gender* | Male=1；Female=2 |
| Age | 18 years and under=1；19 years=2；20 years=3；21 years and older=4 |
| Single son or daughter | Yes=1；No=2 |
| Left-behind experience before 18 years old | Yes=1；No=2 |
| Monthly cost of living | 0 ~ 1000 yuan=1；1001 ~ 1500 yuan=2；  1501 ~ 2000 yuan=3；2001 Yuan and above=4 |
| Family annual income | 0 ~ 30000 Yuan =1；30001 ~ 50000 Yuan =2；50001 ~ 80000 Yuan =3；80001 ~ 100000 Yuan =4；100001 ~ 150000 Yuan =5；150001 Yuan and above =6 |
| Father's educational level | primary school and below=1；junior high school=2；high school=3；college=4；undergraduate=5；master's degree and above=6 |
| Mother's educational level | primary school and below=1；junior high school=2；high school=3；college=4；undergraduate=5；master's degree and above=6 |
| Like own professional | Yes=1；No=2 |
| Will choose own professional again | Yes=1；No=2 |
| Goals after graduation | No direction=1；employment=2；civil servants=3；postgraduate=4；other=5 |

S2 Chinese version PHQ-9

| 在过去的两周里，您感觉以下症状出现的频率有多少？ | 完全没有（0分） | 有几天（1分） | 一半以上的时间（2分） | 几乎每天（3分） |
| --- | --- | --- | --- | --- |
| 1.做事时提不起劲或没有兴趣 | 0 | 1 | 2 | 3 |
| 2.感到心情低落、沮丧或绝望 | 0 | 1 | 2 | 3 |
| 3.入睡困难、睡不安稳或睡眠过多 | 0 | 1 | 2 | 3 |
| 4.感觉疲倦或没有活力 | 0 | 1 | 2 | 3 |
| 5.食欲不振或吃太多 | 0 | 1 | 2 | 3 |
| 6.觉得自己很糟，或感觉自己很失败，或让自己或家人失望 | 0 | 1 | 2 | 3 |
| 7.对事物专注有困难，例如阅读报纸或看电视时不能集中注意力 | 0 | 1 | 2 | 3 |
| 8.动作或说话速度缓慢到别人已经觉察？或正好相反，烦躁或坐立不安、动来动去的情况胜于平常 | 0 | 1 | 2 | 3 |
| 9.有不如死掉或用某种方式伤害自己的念头 | 0 | 1 | 2 | 3 |

S3 Chinese version GAD-7

| 在过去的两周里，您感觉以下症状出现的频率有多少？ | 完全没有（0分） | 有几天（1分） | 一半以上的时间（2分） | 几乎每天（3分） |
| --- | --- | --- | --- | --- |
| 1.感觉紧张、焦虑或烦躁 | 0 | 1 | 2 | 3 |
| 2.不能停止或控制担忧 | 0 | 1 | 2 | 3 |
| 3.对各种各样的事情担忧过多 | 0 | 1 | 2 | 3 |
| 4.很难放松下来 | 0 | 1 | 2 | 3 |
| 5.由于不安而无法静坐 | 0 | 1 | 2 | 3 |
| 6.变得容易烦恼或急躁 | 0 | 1 | 2 | 3 |
| 7.害怕将有可怕的事发生 | 0 | 1 | 2 | 3 |

S4 Chinese version MPAI

| 在过去的两周里，您感觉以下症状出现的频率有多少？ | 完全没有（1分） | 偶尔（2分） | 有时（3分） | 经常（4分） | 总是（5分） |
| --- | --- | --- | --- | --- | --- |
| 1.你的朋友和家人曾因为你在用手机而抱怨 | 1 | 2 | 3 | 4 | 5 |
| 2.有人说过你花了太多的时间在手机上面 | 1 | 2 | 3 | 4 | 5 |
| 3.你曾试图向其他人隐瞒你在手机上花了多长时间 | 1 | 2 | 3 | 4 | 5 |
| 4.你的话费超支 | 1 | 2 | 3 | 4 | 5 |
| 5.你发现自己使用手机的时间比本来打算的要长 | 1 | 2 | 3 | 4 | 5 |
| 6.你尝试在手机上少花些时间但是做不到 | 1 | 2 | 3 | 4 | 5 |
| 7.你从未觉得在手机上花够了时间 | 1 | 2 | 3 | 4 | 5 |
| 8.当手机无信号时,你总担心会错过电话 | 1 | 2 | 3 | 4 | 5 |
| 9.你很难做到将手机关机 | 1 | 2 | 3 | 4 | 5 |
| 10.如果你有一阵子没有査看短信或者手机没有开机,你会变得焦虑 | 1 | 2 | 3 | 4 | 5 |
| 11.没有手机你会心神不定 | 1 | 2 | 3 | 4 | 5 |
| 12.如果你没有手机,你的朋友会很难联系到你 | 1 | 2 | 3 | 4 | 5 |
| 13.当感到被孤立时,你会用手机与别人联系 | 1 | 2 | 3 | 4 | 5 |
| 14.当你感到孤独的时候,你会用手机与别人联系 | 1 | 2 | 3 | 4 | 5 |
| 15.当心情低落的时候,你会玩手机来改善情绪 | 1 | 2 | 3 | 4 | 5 |
| 16.你发现自己在做其他必须要做的事情时却沉迷于手机,为此给你带来些麻烦 | 1 | 2 | 3 | 4 | 5 |
| 17.在手机上耗费的时间直接导致你的办事效率降低 | 1 | 2 | 3 | 4 | 5 |

S5 Chinese version AMS

| 题目 | 完全不符合（1分） | 有些不符合（2分） | 基本符合（3分） | 非常符合（4分） |
| --- | --- | --- | --- | --- |
| 1. 我喜欢新奇的、有困难的任务，甚至不惜冒风险 | 1 | 2 | 3 | 4 |
| 2. 我在完成有困难的任务时，感到快乐 | 1 | 2 | 3 | 4 |
| 3. 我会被那些能了解自己有多大才智的工作所吸引 | 1 | 2 | 3 | 4 |
| 4. 我喜欢尽了最大努力能完成的工作 | 1 | 2 | 3 | 4 |
| 5. 我喜欢对我没有把握解决的问题坚持不懈地努力 | 1 | 2 | 3 | 4 |
| 6. 对于困难的任务，即使没有什么意义，我也很容易 卷进去 | 1 | 2 | 3 | 4 |
| 7. 面对能测量我能力的机会，我感到是一种鞭策和挑 战 | 1 | 2 | 3 | 4 |
| 8. 我会被有困难的任务所吸引 | 1 | 2 | 3 | 4 |
| 9. 对于那些我不能确定是否能成功的工作，最能吸引 我 | 1 | 2 | 3 | 4 |
| 10. 给我的任务即使有充裕的时间，我也喜欢立即开始 做 | 1 | 2 | 3 | 4 |
| 11.能够测量我能力的机会，对我是有吸引力的 | 1 | 2 | 3 | 4 |
| 12. 面临我没有把握克服的难题时，我会非常兴奋，快 乐 | 1 | 2 | 3 | 4 |
| 13. 如果有事不能立刻理解，我会很快对它产生兴趣 | 1 | 2 | 3 | 4 |
| 14. 对我来说，重要的是做有困难的事，即使无人知道 也无关重要 | 1 | 2 | 3 | 4 |
| 15. 我希望把有困难的工作分配给我 | 1 | 2 | 3 | 4 |
| 16. 我讨厌在完全不能确定会不会失败的情境中工作 | 1 | 2 | 3 | 4 |
| 17. 在结果不明的情况下，我担心失败 | 1 | 2 | 3 | 4 |
| 18. 在完成我认为是有困难的任务时，我担心失败 | 1 | 2 | 3 | 4 |
| 19. 一想到要去做那些新奇的、有困难的工作，我就感到 不安 | 1 | 2 | 3 | 4 |
| 20. 我不喜欢那些测量我能力的场面 | 1 | 2 | 3 | 4 |
| 21. 我对那些没有把握能胜任的工作感到忧虑 | 1 | 2 | 3 | 4 |
| 22. 我不喜欢做我不知道能否完成的事，即使别人不知 道也一样 | 1 | 2 | 3 | 4 |
| 23. 在那些测量能力的情境中，我感到不安 | 1 | 2 | 3 | 4 |
| 24. 对需要有特定机会才能解决的事，我会害怕失败 | 1 | 2 | 3 | 4 |
| 25. 那些看起来相当困难的事，我做时很担心 | 1 | 2 | 3 | 4 |
| 26. 我不喜欢在不熟悉的环境下工作，即使无人知道也 一样 | 1 | 2 | 3 | 4 |
| 27. 如果有困难的工作要做，我希望不要分配给我 | 1 | 2 | 3 | 4 |
| 28.我不希望做那些要发挥我能力的工作 | 1 | 2 | 3 | 4 |
| 29. 我不喜欢做那些我不知道我能否胜任的事 | 1 | 2 | 3 | 4 |
| 30. 当我遇到我不能立即弄懂的问题，我会焦虑不安 | 1 | 2 | 3 | 4 |

S6 Scale reliability and validity analysis

| Scale | Standardized α | KMO | Bartlett's Test of Sphericity | | |
| --- | --- | --- | --- | --- | --- |
|  |  |  | χ² | df | p |
| PHQ-9 | 0.858 | 0.890 | 8533.810 | 36 | **<0.001** |
| GAD-7 | 0.898 | 0.914 | 10042.249 | 21 | **<0.001** |
| MPAI | 0.883 | 0.880 | 18797.680 | 136 | **<0.001** |
| AMS | 0.767 | 0.943 | 34426.317 | 435 | **<0.001** |

S7 Scale reliability and validity analysis of PHQ-9

| Item No. | Cronbach’α | Standardized Loading | AVE |
| --- | --- | --- | --- |
| 1 | 0.858 | 0.744 | **0.889** |
| 2 |  | 0.733 |  |
| 3 |  | 0.667 |  |
| 4 |  | 0.769 |  |
| 5 |  | 0.698 |  |
| 6 |  | 0.745 |  |
| 7 |  | 0.670 |  |
| 8 |  | 0.634 |  |
| 9 |  | 0.502 |  |

S8 Scale reliability and validity analysis of GAD-7

| Item No. | Cronbach’α | Standardized Loading | AVE |
| --- | --- | --- | --- |
| 1 | 0.898 | 0.773 | **0.920** |
| 2 |  | 0.848 |  |
| 3 |  | 0.818 |  |
| 4 |  | 0.826 |  |
| 5 |  | 0.778 |  |
| 6 |  | 0.777 |  |
| 7 |  | 0.695 |  |

S9 Scale reliability and validity analysis of MPAI

| Dimension | Item No. | Cronbach’α | Standardized Loading | AVE |
| --- | --- | --- | --- | --- |
| Loss of Control | 1 | 0.780 | 0.838 | **0.713** |
|  | 2 |  | 0.840 |  |
|  | 3 |  | 0.632 |  |
|  | 4 |  | 0.357 |  |
|  | 5 |  | 0.308 |  |
|  | 6 |  | 0.269 |  |
|  | 7 |  | 0.226 |  |
| Withdrawal | 8 | 0.786 | 0.725 | **0.801** |
|  | 9 |  | 0.670 |  |
|  | 10 |  | 0.769 |  |
|  | 11 |  | 0.664 |  |
| Escapism | 12 | 0.754 | 0.509 | **0.785** |
|  | 13 |  | 0.813 |  |
|  | 14 |  | 0.872 |  |
| Inefficiency | 15 | 0.757 | 0.672 | **0.800** |
|  | 16 |  | 0.769 |  |
|  | 17 |  | 0.821 |  |

Note: Although the standardized loading of some items are less than 0.4, they are retained to maintain the integrity of the original scale.

S10 Scale reliability and validity analysis of AMS

| Dimension | Item No. | 成分 | Cronbach’α | Standardized Loading | AVE |
| --- | --- | --- | --- | --- | --- |
| MS | 1 | 5 | 0.889 | -0.515 | **0.894** |
|  | 2 | 3 |  | 0.720 |  |
|  | 3 | 3 |  | 0.726 |  |
|  | 4 | 3 |  | 0.691 |  |
|  | 5 | 2 |  | 0.487 |  |
|  | 6 | 2 |  | 0.593 |  |
|  | 7 | 3 |  | 0.613 |  |
|  | 8 | 2 |  | 0.588 |  |
|  | 9 | 2 |  | 0.696 |  |
|  | 10 | 5 |  | 0.620 |  |
|  | 11 | 3 |  | 0.606 |  |
|  | 12 | 2 |  | 0.725 |  |
|  | 13 | 2 |  | 0.601 |  |
|  | 14 | 2 |  | 0.718 |  |
|  | 15 | 2 |  | 0.723 |  |
| MF | 16 | 4 | 0.911 | 0.606 | **0.926** |
|  | 17 | 4 |  | 0.779 |  |
|  | 18 | 4 |  | 0.699 |  |
|  | 19 | 1 |  | 0.591 |  |
|  | 20 | 1 |  | 0.641 |  |
|  | 21 | 1 |  | 0.663 |  |
|  | 22 | 1 |  | 0.676 |  |
|  | 23 | 1 |  | 0.723 |  |
|  | 24 | 1 |  | 0.673 |  |
|  | 25 | 1 |  | 0.674 |  |
|  | 26 | 1 |  | 0.673 |  |
|  | 27 | 1 |  | 0.690 |  |
|  | 28 | 1 |  | 0.647 |  |
|  | 29 | 1 |  | 0.716 |  |
|  | 30 | 1 |  | 0.643 |  |

S11 Post hoc LSD or Tamhane's significance

| Items | Options | homogeneity of variance | F | P | Post hoc  LSD or Tamhane's significance | | | | |
| --- | --- | --- | --- | --- | --- | --- | --- | --- | --- |
|  |  |  |  |  | Options | Mean difference (I-J) | SE | P | 95 % CI |
| Family annual income | 0 ~ 30000 Yuan | No | 6.340 | ＜0.001 | 10001~30000 Yuan vs. 100001~150000 Yuan | -2.40566 | 0.72715 | **0.014** | (-4.5394, -0.2719) |
|  | 30001 ~ 50000 Yuan |  |  |  | 10001~30000 Yuan vs. 150001 Yuan and above | -3.68792 | 0.84388 | **<0.001** | (-6.1656, -1.2102) |
|  | 50001 ~ 80000 Yuan |  |  |  | 30001~50000 Yuan vs. 100001 ~ 150000 Yuan | -2.24146 | 0.74724 | **0.041** | (-4.4347, -0.0482) |
|  | 80001 ~ 100000 Yuan |  |  |  | 30001~50000 Yuan vs. 150001 Yuan and above | -3.52372 | 0.86126 | **0.001** | (-6.0526, -0.9948) |
|  | 100001 ~ 150000 Yuan |  |  |  | 50001~80000 Yuan vs. 150001 Yuan and above | -3.20755 | 0.93619 | **0.010** | (-5.9573, -0.4578) |
|  | 150001 Yuan and above |  |  |  | 10001~30000 Yuan vs. 100001 ~ 150000 Yuan | -2.40566 | 0.72715 | **0.014** | (-4.5394, -0.2719) |
| Father's educational level | primary school and below | Yes | 3.236 | 0.006 | junior high school vs. undergraduate | -2.61544 | 0.71609 | **0.004** | (-4.7179, -0.5129) |
|  | junior high school |  |  |  |  |  |  |  |  |
|  | high school |  |  |  |  |  |  |  |  |
|  | college |  |  |  |  |  |  |  |  |
|  | undergraduate |  |  |  |  |  |  |  |  |
|  | master's degree and above |  |  |  |  |  |  |  |  |
| Mother's educational level | primary school and below | Yes | 3.634 | 0.003 |  |  |  |  |  |
|  | junior high school |  |  |  | primary school and below vs. junior high school | -2.27963 | 0.74023 | **0.002** | (-3.7311, -0.8281) |
|  | high school |  |  |  | primary school and below vs. high school | -1.65523 | 0.83332 | **0.047** | (-3.2893, -0.0212) |
|  | college |  |  |  | primary school and below vs. college | -2.00238 | 0.83811 | **0.017** | (-3.6458, -0.3590) |
|  | undergraduate |  |  |  | primary school and below vs. undergraduate | -3.59699 | 0.88294 | **0.000** | (-5.3283, -1.8657) |
|  | master's degree and above |  |  |  |  |  |  |  |  |
| Goals after graduation | No direction | No | 6.137 | ＜0.001 |  |  |  |  |  |
|  | employment |  |  |  | employment vs. postgraduate | -2.85136 | 0.77962 | **0.003** | (-5.0482, -0.6545) |
|  | civil servants |  |  |  | postgraduate vs. employment | 2.85136 | 0.77962 | **0.003** | (0.6545, 5.0482) |
|  | postgraduate |  |  |  |  |  |  |  |  |
|  | other |  |  |  |  |  |  |  |  |
